# Identification of water sources of mine water bursts based on the FPS-DT model

**DOI:** 10.1038/s41598-025-13301-y

**Published:** 2025-07-27

**Authors:** Kaide Liu, Yu xia, Xiaolong Li, Chaowei Sun, Wenping Yue, Qiyu Wang, Songxin Zhao, Shufeng Chen

**Affiliations:** 1 Shaanxi Key Laboratory of Safety and Durability of Concrete Structures, Xijing University, Xi’an, 710123 China; 2 Xi’an Research Institute, China Coal Technology and Engineering Group Corp, Xi’an, 710077 China

**Keywords:** Principal component analysis (PCA), Fuzzy C-Means, SMOTE oversampling, Decision tree, Hydrochemical characteristics, Environmental sciences, Hydrology, Energy science and technology, Engineering

## Abstract

To effectively identify the source of water in coal mines and prevent water-related accidents, this paper utilises the hydrochemical characteristics of the aquifers Shanxi Hanzui Coal Mine. The fuzzy C-means (FCM) clustering method is employed to classify water sample data, followed by principal component analysis (PCA) for dimensionality reduction to extract key features. The SMOTE algorithm is then applied to address the issue of class imbalance. Based on this, a decision tree model (FPS-DT) is constructed using the CART algorithm. To validate the model’s performance, five-fold cross-validation was used for evaluation. The results showed that the average classification accuracy of the FPS-DT model was 93%. In contrast, the accuracy of the comparison model, which only used PCA and decision trees, was 78%, indicating that the method proposed in this paper has significant advantages in terms of identification accuracy and generalisation capability. Additionally, the FPS-DT model features a clear structure and explicit classification rules, offering good interpretability and robustness. It can adapt to the real-time water source identification requirements of complex underground environments, providing theoretical support and technical assurance for coal mine safety production and water hazard prevention and control.

## Introduction

China possesses abundant coal resources, with proven coal reserves reaching 218.57 billion tonnes by the end of 2023. The country has been mining over 3.5 billion tonnes annually^1^solidifying coal’s crucial role in its energy mix^2^. However, the exploitation of these resources has long been hindered by water hazards. As mining operations progress to greater depths in recent years, the complexity and diversity of water hazards have escalated. Consequently, the challenges associated with flood prevention and control have intensified, leading to frequent mine flooding accidents that result in significant injuries, fatalities, and economic losses^3^. One such incident occurred in March 2010 at the Wangjialing coal mine, where water accumulation in the mining void caused a leakage^4^. This disaster trapped 153 miners and claimed 38 lives. These events underscore the urgent need for effective and timely water damage prevention strategies. A critical prerequisite for such strategies is the rapid and accurate identification of potential water surge sources, which forms the foundation for efficient mine water control measures^5–7^.

Currently, several methods have been proposed for mine w ater source identification, including chemical analysis, hydrodynamic analysis, water temperature analysis, water level dynamic observation, and geophysical exploration, among others^8–13^. Among these, the chemical analysis method is commonly used to identify sudden water sources by analyzing the chemical characteristics of the water. This method is particularly convenient, rapid, and widely employed, making it the most frequently adopted approach by researchers^14^.ZHU Saijun^15^ proposed a model for identifying sudden mine water sources, which is Identification of mine water inrush source based on combination weight-theory of improved grey relational degree, Despite its high accuracy and applicability, the model requires high-quality and complete data. Additionally, when two sets of data are highly similar in feature space, the model exhibits uncertainty in distinguishing the boundaries between neighboring samples, which may negatively impact the accuracy and reliability of the classification results. Feng Dongmei and Wu Jianwei^16^ developed a correlation theory-driven hybrid model integrating Support Vector Machine (SVM) algorithms for discrimination of mine water inrush sources. Although this methodology demonstrates enhanced robustness in handling multi-indicator systems with inherent interdependencies, its efficacy in resolving multiclass discrimination tasks remains suboptimal, primarily due to the inherent limitations of binary classification frameworks in SVM architectures when extrapolated to high-dimensional categorical scenarios. By integrating the centroid distance metric with Fisher discriminant analysis (FDA), Zhao, W. et al.^17^ and Sun, F. X. et al.^18^ successfully incorporated the evaluation of centroid distances into Fisher’s discriminant analysis for water source identification. This method significantly enhanced the accuracy of classification, demonstrating its effectiveness in classifying water chemistry characteristics.However, this method is more sensitive to outliers, requiring rigorous pre-processing (e.g., standardization or normalization) of the data during practical applications to mitigate the impact of magnitude differences during distance calculations ; Furthermore, since the center-of-mass distance method fails to appropriately account for the varying importance of features, the issue of feature weighting remains unresolved, which indicates a higher likelihood of different chemical features influencing the balance of classificatory contributions.

Although the above methods play a significant role in advancing water source identification, particularly in the context of mine water bursts, they face challenges such as the imbalance of sample data, which makes it difficult for discriminative models to comprehensively capture the features of different categories; furthermore, when dealing with neighboring samples that exhibit high similarity, their boundaries become challenging to accurately define, thereby increasing classification uncertainty; Additionally, in multiclassification tasks, existing methods have insufficient refinement in handling complex category structures, which limits the recognition accuracy of the models. More importantly, many models do not allow for intuitive visualization of the process, which imposes limitations on both the interpretability of the results and the operational efficiency of practical applications. Therefore, in-depth research and improvement for these issues are of great scientific significance and application value. Especially important is optimising the sample distribution, enhancing the model’s ability to identify complex boundaries, improving multi-classification task processing accuracy, as well as increasing visualization and interpretability of models.

This study proposes a novel hybrid approach integrating Principal Component Analysis (PCA), Fuzzy C-means Clustering (FCM), and Synthetic Minority Oversampling Technique (SMOTE)-enhanced Decision Tree (DT) to address data complexity and class imbalance challenges. The framework leverages FCM for fuzzy pattern recognition, PCA for dimensionality reduction, SMOTE for minority class augmentation, and DT for interpretable classification, achieving balanced performance in high-dimensional imbalanced scenarios.

Viewing the above analysis, this article proposes an effective combination of a decision tree (DT) algorithm based on Principal Component Analysis (PCA), which integrates fuzzy C-mean clustering and SMOTE oversampling techniques to address the challenges in classifying water sources. This study focuses on water samples from the 2# coal seam old empty well and bedded sandrock fracture water in Xiangning Mine. Through system analysis of the water sample data, it was found that the aforementioned ions—sodium (Na^+^) and potassium (K^+^), calcium (Ca^2+^), magnesium (Mg^2+^), chloride (Cl^−^), sulfate (SO_4_^2−^), bicarbonate (HCO_2_−), and carbonate (CO_3_^2−^)—play a significant role in feature recognition. After preprocessing the data by addressing missing values, outliers, and duplicate data points, we obtained complete datasets. Applying FCM clustering analysis enables the identification of water sample types based on the obtained cluster centers. PCA is employed to reduce dimensionality and eliminate redundant features, thereby simplifying the model structure. SMOTE over-sampling technique is utilized to balance sample categories^19^addressing class imbalance issues and enhancing the generalization capability of decision trees. Ultimately, through decision tree CART algorithm prediction training using the trained model on the test set, the generalization ability of the model was assessed to optimize model parameters for accurate judgment of source of inrush water. Based on reasonable feature selection and data balancing techniques, the decision tree model demonstrates high classification accuracy. Additionally, The decision tree model offers both high interpretability and strong robustness^20–22^making it well-suited for complex classification tasks involving environmental or hydrochemical data. By transforming decision processes into a binary tree structure—where each internal node represents a feature-based splitting rule and each leaf node corresponds to a predicted class—the model allows for full traceability of the decision pathway. This hierarchical structure enables researchers to visualize and interpret the model’s internal logic, thereby facilitating validation of the classification outcomes and ensuring transparency in model reasoning. In addition to interpretability, decision trees exhibit inherent robustness to outliers, owing to their use of impurity-based splitting criteria, such as the Gini index, which minimize the influence of extreme values on the overall tree structure. During the recursive partitioning process, the algorithm consistently prioritizes features with the highest discriminative power, thereby reducing the impact of irrelevant or noisy attributes. Furthermore, when applied in a reduced-dimensional principal component space, decision trees are capable of capturing localized nonlinear decision boundaries, leading to improved classification accuracy while preserving model simplicity and computational efficiency. This study presents a novel method for identifying water inrush sources in mines and contributes to the prevention and control of mine water hazards^23^.

## Methods

### Fuzzy C-mean clustering algorithm

Fuzzy C-Means (FCM) is a widely used fuzzy clustering algorithm in data cluster analysis^24–26^. It clusters samples based on their Euclidean distances from the clusters and their memberships. Unlike K-means is a classical hard clustering algorithm that partitions data by minimizing the Euclidean distance between each sample and its assigned cluster center^27^. While K-means performs efficiently on datasets with well-separated, compact clusters and relatively uniform distributions—such as those found in image compression or market segmentation—its effectiveness diminishes when handling overlapping classes or transitional data, FCM allows each data point to belong to multiple clusters with varying membership degrees based on its characteristics. This characteristic makes FCM particularly effective in handling situations involving uncertainty or ambiguity, offering a more flexible and adaptable approach to clustering. The objective of the FCM algorithm is to minimize a cost function, which is the sum of the weighted distances between data points and cluster centers, along with the affiliation matrix. The algorithm iteratively updates both the cluster centers and membership values to achieve an optimal data partition. Through this fuzzy partitioning, FCM can uncover the underlying structure of the data, especially in cases where cluster boundaries are not clearly defined, thereby providing more detailed and nuanced clustering results.

Such a mechanism is particularly advantageous in the context of hydrochemical datasets, where transitions between water types are often continuous rather than discrete. The capacity of FCM to model this gradual change enables a more realistic representation of the inherent ambiguity in water source classification. Moreover, FCM minimizes a membership-weighted objective function based on the distances between samples and cluster centroids, thereby enhancing its robustness against noise and minor fluctuations in the data^28^.The objective function J_m_ is defined as:

1 $$J_{m} = \sum\limits_{{i = 1}}^{N} {\sum\limits_{{j = 1}}^{C} {u_{{ij}}^{m} } } \cdot \left\| {x_{i} - c_{j} } \right\|^{2}$$

Where N is the number of samples, C is the number of clusters, u_ij_ represents the membership degree of the i-th sample point to the j-th cluster, m is the fuzziness factor, typically set as m > 1, which controls the degree of fuzziness in the membership, x_i_ is the feature vector of the i-th sample, and c_j_ is the center of the j-th cluster.

the basic procedure of the FCM algorithm for clustering is as follows:

1. Initialize the membership matrix: randomly initialize the membership matrix *U*
2. Update cluster centers: Based on the current membership matrix U, update the center of each cluster c_j_:
  2 $${c_j}=\frac{{\sum\limits_{{i=1}}^{N} {u_{{ij}}^{m}} \cdot {x_i}}}{{\sum\limits_{{i=1}}^{N} {u_{{ij}}^{m}} }}$$
3. Update the membership matrix: Using the new cluster centers, recalculate the membership matrix U, where the membership uij is adjusted according to the distance between the sample point and each cluster center:
  3 $$u_{{ij}} = \frac{1}{{\sum\limits_{{k = 1}}^{C} {\left( {\frac{{\left\| {x_{i} - c_{j} } \right\|}}{{\left\| {x_{i} - c_{k} } \right\|}}} \right)^{{\frac{2}{{m - 1}}}} } }}$$
4. Repeat until convergence: Repeat steps 2 and 3 until the objective function Jm converges to a predefined threshold or the maximum number of iterations is reached.

Clustering the data allows us to group seemingly irregular data into distinct clusters based on their similarity. This process enables the classification of data into different types, followed by labeling the data according to these clusters. These cluster labels can then be used as features to assist classifiers in more effectively categorizing the data, ultimately improving prediction accuracy.

### Principal component analysis

Principal Component Analysis (PCA) is a fundamental technique in multivariate statistics^19,29–31^employed to simplify datasets by projecting the original data onto a new coordinate system through linear transformation. This method preserves the most significant features of the data, reducing its dimensionality while retaining as much of the key information as possible. Principal Component Analysis (PCA) is extensively employed in data preprocessing, dimensionality reduction, visualization, and feature extraction. The fundamental principle of PCA involves projecting the original data onto a new set of mutually orthogonal basis vectors, termed principal components, through a linear transformation. These principal components are ordered according to the magnitude of data variance: the first principal component captures the direction of maximum variance, the second principal component represents the direction of the largest variance within the remaining data, and so forth. The steps to perform Principal Component Analysis (PCA) are as follows:

1. **Standardization**: First, calculate the mean and standard deviation of each feature, then convert the data into a standard normal distribution with a mean of 0 and a standard deviation of 1. The standardization is expressed as:

4 $$Z=\frac{{X - \mu }}{\sigma }$$

where X is the original data, µ is the mean, and σ is the standard deviation.

(2) **Covariance matrix calculation**: Next, compute the covariance matrix from the standardized data matrix Z:
  5 $$\Sigma =\frac{1}{{n - 1}}{Z^T}Z$$
(3) **Eigenvalue and eigenvector calculation**: Finally, derive the eigenvalues of the covariance matrix Σ, and select the eigenvectors corresponding to the largest eigenvalues as the principal components. Typically, the components with a cumulative variance contribution greater than or equal to 85% are chosen as the principal components (Ju and Hu, 2021). The formula for cumulative variance contribution is as follows:
  6 $$C=\frac{{\sum\limits_{{i=1}}^{k} {{\lambda _i}} }}{{\sum\limits_{{j=1}}^{m} {{\lambda _j}} }}$$

Where C is the cumulative variance contribution, k is the number of selected principal components, and m is the total number of eigenvalues. The principal component samples selected in this manner are sufficient to meet the requirements of the majority of experiments.

### The SMOTE algorithm

The Synthetic Minority Over-sampling Technique (SMOTE) is an oversampling algorithm designed to mitigate class imbalance in classification tasks^32–36^. As an improvement over traditional random oversampling methods, SMOTE generates synthetic samples for the minority class by interpolating between existing minority samples in the feature space. Specifically, for each minority class sample, SMOTE identifies its k nearest neighbors (K-NN) and creates new synthetic samples along the line segments connecting the sample to randomly selected neighbors. This process effectively increases the representation of the minority class, thereby shifting the decision boundary closer to the majority class. By introducing greater diversity among the minority samples, SMOTE helps to alleviate overfitting and substantially enhances the generalization capability of the classifier.

In this study, we apply the SMOTE algorithm to augment the minority class and rectify the sample imbalance within our dataset. The detailed algorithmic procedure is as follows:

1. For each sample $\:{X}_{i}$ in the minority class, calculate the Euclidean distance as the criterion, denoted by:

7 $$D({x_i},{x_j})=\sqrt {\sum\limits_{{m=1}}^{p} {{{({x_{im}} - {x_{jm}})}^2}} }$$

where $\:{x}_{im}$ and $\:{x}_{jm}$represent the m-th feature of samples $\:{x}_{i}$ and $\:{x}_{j}$, respectively, and p is the number of features. The distance between $\:{X}_{i}$ and all other samples in the minority class dataset is computed to identify the K-nearest neighbors. Let $\:{X}_{i}$ be a sample from the minority class and $\:{N}_{i}$ the set of its K-nearest neighbors, such that $N_{i} = \left\{ {X_{{i1}} ,\,X_{{i2}} ,....X_{{ik}} } \right\}$

(2) Randomly select a neighbor $\:{X}_{ni}$ from $\:{N}_{i}$ and compute the new synthetic sample using the following formula:

8 $${X_{new}}={X_i}+\delta \cdot ({X_{ni}} - {X_i})$$

where δ is a random number in the range [0, 1].

(3) Repeat the above steps until the desired number of minority class samples are generated.

**Fig. 1:**
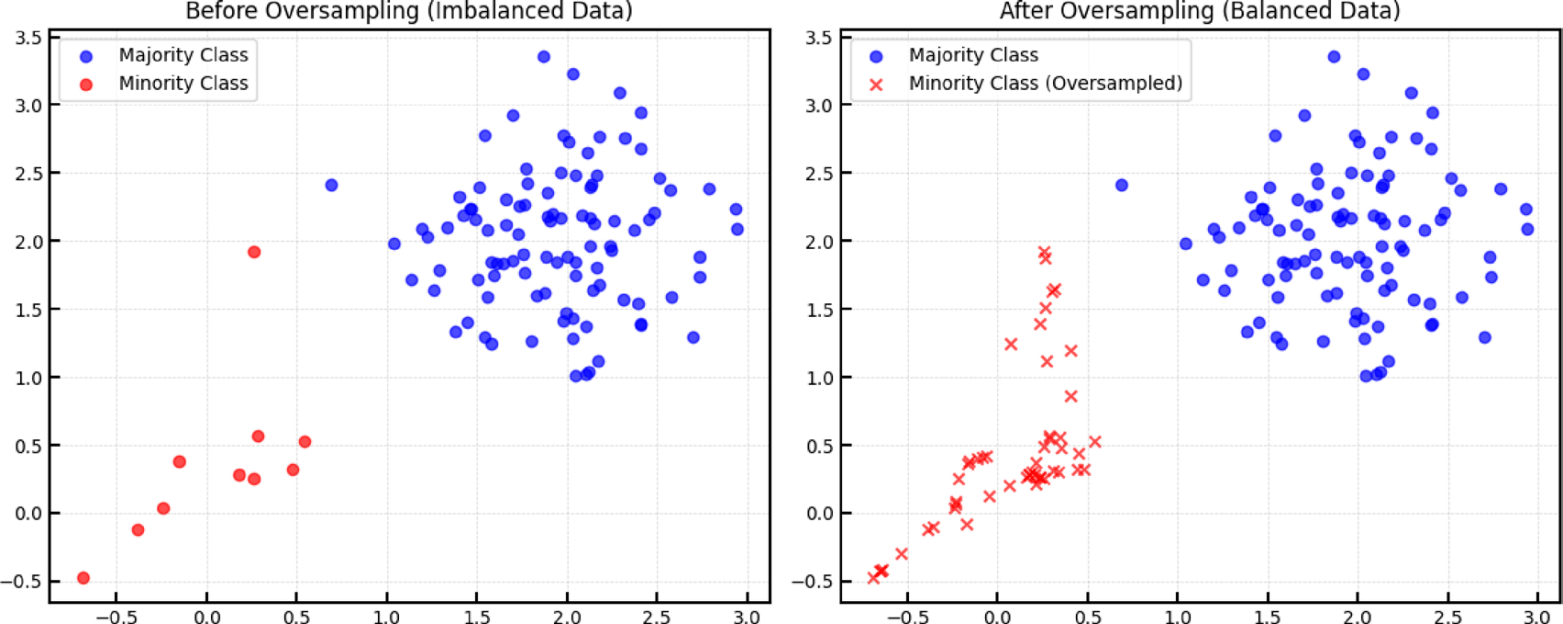
Explanation of over-sampling principle.

By addressing class imbalance, SMOTE enables the model to better recognize patterns and generalize across classes, thereby enhancing overall performance. It helps mitigate the bias introduced by class imbalance by ensuring the model is not overly influenced by the majority class at the expense of the minority class(Fig. 1). This approach effectively increases the number of samples in the minority class without the need for additional data collection, making it a resource-efficient technique.

### Decision tree algorithms

Decision Tree (DT) is a tree-structured machine learning algorithm^37–41^ commonly used for classification and regression tasks. Decision tree algorithms are favored for their simplicity, interpretability, and computational efficiency, which is why they are widely applied in various classification and recognition domains. A decision tree works by recursively partitioning a dataset into smaller subsets based on a series of rules, while organizing decisions into a tree structure. Each internal node represents a decision based on a feature, each branch corresponds to the outcome of the decision, and each leaf node represents the final classification or regression result. The primary advantage of decision trees lies in their ability to break down a complex decision-making process into a sequence of straightforward judgment steps, constructing a model that is easy to interpret. The Classification and Regression Tree (CART) algorithm employed in this study constructs binary decision trees by selecting optimal feature splits based on the Gini index, which serves as the impurity measure. This criterion aims to maximize class separation while minimizing heterogeneity within each node^42^.

In this study, the classification model was constructed using the Classification and Regression Tree (CART) algorithm, a decision tree method particularly well-suited for classification tasks. One of the key advantages of CART lies in its interpretable, rule-based structure, which recursively partitions the dataset based on feature values to form a binary tree. The core mechanism of CART involves selecting, at each node, the optimal feature and corresponding split point that produce the most homogeneous child nodes.To achieve this, the CART algorithm employs Gini impurity as the splitting criterion. Gini impurity is a measure of the degree of class heterogeneity within a dataset (or node) and serves as an indicator of node purity. For a given dataset D, the Gini impurity is computed using the following formula:

9 $$Gini(D)=1 - \sum\limits_{{i=1}}^{n} {{{({p_i})}^2}}$$

Where $\:D$ represents the dataset, $\:n$ denotes the number of classes, and $\:{p}_{i}$ represents the proportion of samples belonging to class $\:i$ within the dataset.

The significance is to reflect the probability of randomly extracting two samples from binary tree nodes to belong to different categories. The smaller the Gini value (approaching 0), the higher the node purity and meaning all samples within the node belong to a single class.Conversely, higher Gini impurity values reflect greater class heterogeneity within the node, implying increased uncertainty in classification at that point in the tree.

The feature selection process is executed at each node of the decision tree. Initially, all 42 samples are grouped at the root node. For each candidate feature $\:{x}_{j}$,the Gini impurity is calculated, and all possible split thresholds s within the range of $\:{x}_{j}$ are enumerated to identify the optimal split point. For each candidate pair ($\:{x}_{j}$,s)sthe current dataset D is partitioned into two subsets, D_1_ and D_2_, according to whether the feature values satisfy the splitting condition:

$$D_{1} = \{ x \in D|x_{j} \le s\} ,\,D_{2} = \{ x \in D|x_{j} > s\}$$

Subsequently, the weighted average Gini impurity corresponding to the split is computed, serving as a metric to evaluate the overall impurity of the resulting child nodes. This metric reflects the degree of class homogeneity achieved by the split and is used to assess its effectiveness:

10 $$Gini(D,{x_i},s)=\frac{{|{D_1}|}}{{|D|}}Gini({D_1})+\frac{{|{D_2}|}}{{|D|}}Gini({D_2})$$

The Gini impurities of the subsets D_1_ and D_2_ are computed according to Eq. (9), which defines the impurity measure used to evaluate the quality of the split.

At each decision node, the CART algorithm exhaustively evaluates all candidate features $\:{x}_{j}$ and their corresponding split points s to identify the optimal split($\:{x}_{j}$,s) that minimizes the impurity measure Gini(D, $\:{x}_{j}$ ,s). The selected split is then applied to partition the dataset into two child nodes, forming a binary tree structure. This splitting process is recursively repeated for each child node until a stopping condition is met—such as reaching a predefined maximum tree depth, a minimum number of samples per leaf node, or when no further reduction in impurity can be achieved.

**Fig. 2:**
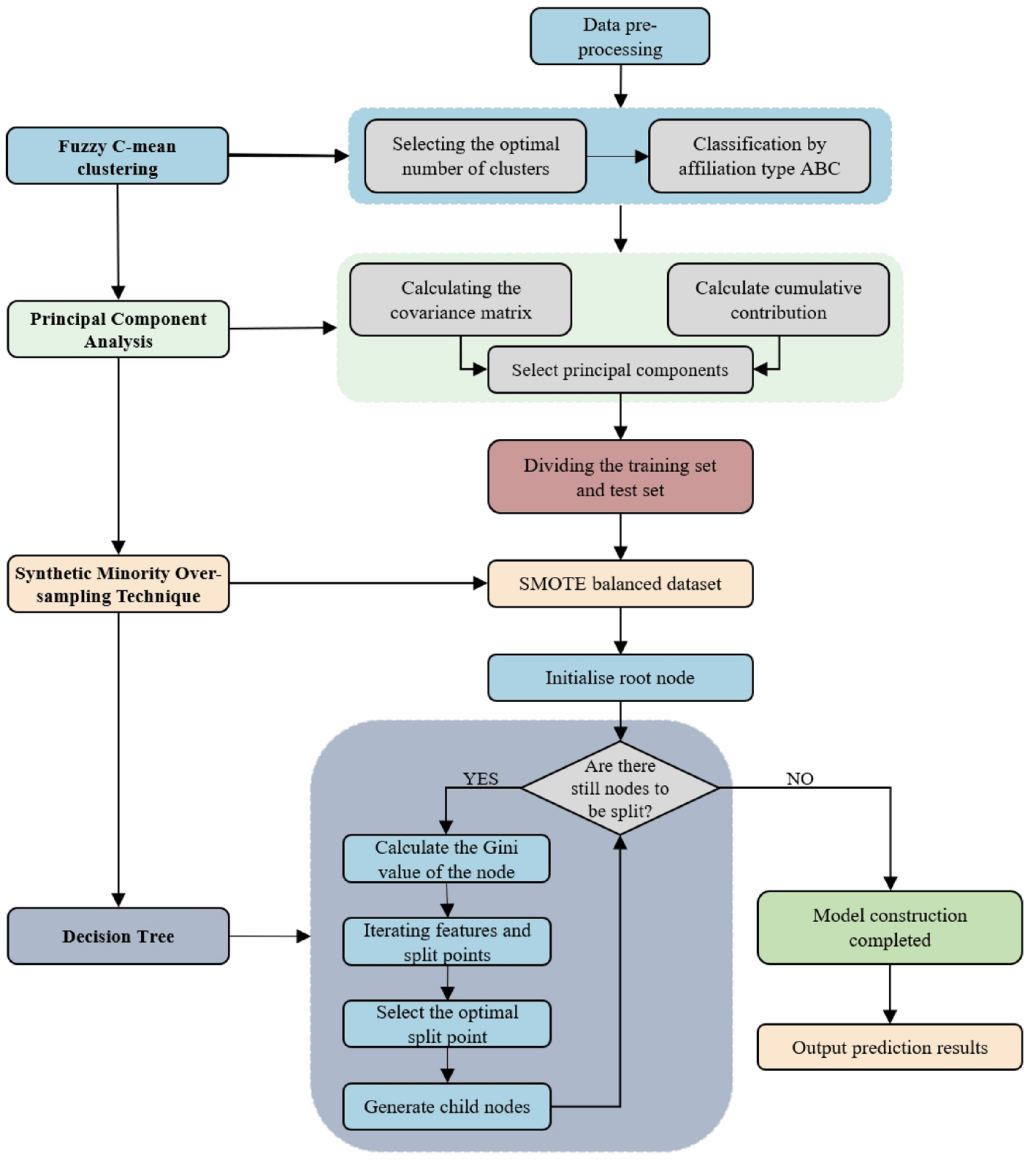
Flowchart based on FPS-DT model.

By recursively selecting the feature and threshold that yield the greatest improvement in node purity, the CART algorithm constructs a decision tree that achieves strong classification performance while maintaining interpretability. The construction process of this FPS-DT model is shown in Fig. 2.

## Analysis of water samples

### Overview of the study area

**Fig. 3:**
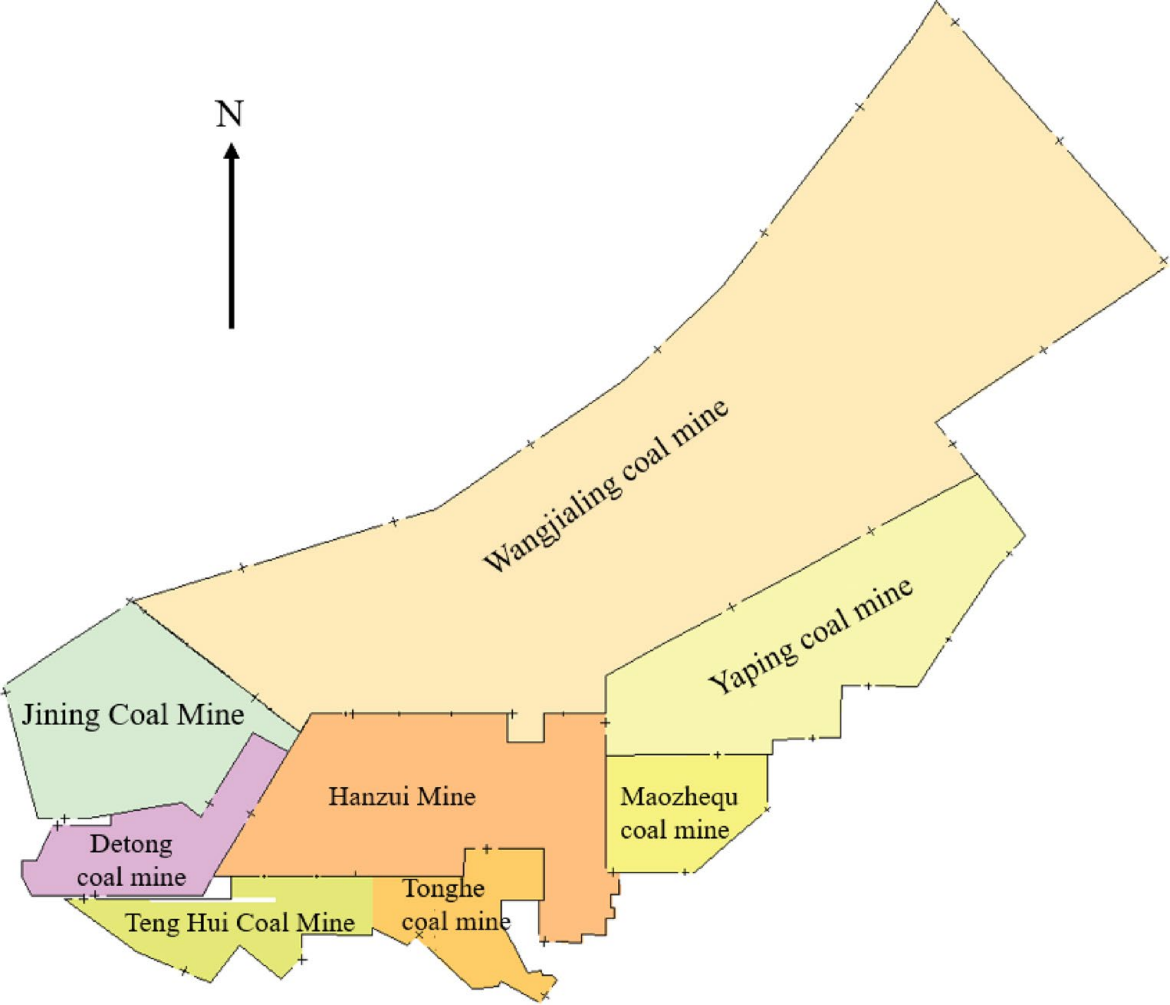
Distribution of coal mines in Xiangning mining area.

The Shanxi Hanzui Coal Mine is situated in the southern part of the Wangjialing Precision Exploration Area, within the Xiangning Mining District of the Hedong Coal Field, as depicted in Fig. 3. The well field lies at the southernmost edge of the Lvliang Mountain range, characterized by high mountains and deep ravines, with a complex topography. The region has a general east-to-west slope, with higher elevations in the east and lower in the west, and is part of the Loess Plateau Zone. The surface water in the mine area belongs to the Yellow River Basin. A key feature of the area is the Anli River, which runs east to west along the northern boundary of the mine. This seasonal river flows out of the area to the west and eventually converges with the Yellow River. The main aquifers in the well field, listed from top to bottom, include: the aquifer in the Quaternary gravel layer with pore-dividing characteristics, the aquifer formed by Permian clastic fissures, the aquifer in the Upper Carboniferous Taiyuan Formation limestone characterized by karst fissures, and the aquifer in the Middle Ordovician formed by karst fissures^43^. Additionally, the primary aquifers in the region, in order, are: the aquifer at the base of the Cenozoic boundary, the aquifer within the Carboniferous diabase layers, and the aquifer of the Taiyuan Formation extending from the coal basement to the upper boundary of the Benxi Formation. The geological conditions of the mining area are complex, with abundant groundwater resources. As coal mining progresses, water gradually accumulates in the mining area, resulting in the formation of old void water and fissure water. These old void waters not only directly affect the water quality of the mining area but also present a potential threat to the regional ecological balance. The possible presence of pollutants within the void water and their interaction with surrounding water bodies further elevates the importance of this issue. Consequently, understanding the water quality characteristics of old void water and its impact on the ecological environment of the mining area is critical for the sustainable development of the region and the integrated management of water resources and ecological protection^44,45^.

### Water quality analysis of Hanzui coal mine

To construct a water source identification model based on 42 data sets of old void water quality from the Hanzui coal mine in Shanxi, a thorough analysis of the water quality at the data collection site is required as the first step(Table 1).

**Table 1:** Water samples from Hanzui coal mine.

Sample number	Na^+^+ K^+^	Ca^2+^	Mg^2+^	Cl^-^	SO_4_^2-^	HCO_3_^-^+CO_3_^2-^	TDS	PH	Water sample type
1	338.40	63.79	26.60	60.93	248.46	859.38	1167.87	7.90	I
2	446.90	29.90	18.14	144.01	219.79	883.48	1303.46	8.06	I
3	563.00	8.12	3.69	57.15	58.40	1330.78	1375.10	7.91	II
4	563.00	8.12	1.23	50.01	51.10	1270.46	1322.08	7.94	II
5	386.10	58.83	28.25	53.72	340.75	851.48	1302.30	7.92	I
6	582.90	12.18	0.00	56.50	19.47	1377.93	1397.35	8.17	II
7	371.50	33.00	31.02	59.50	268.98	959.32	1243.66	7.11	I
8	365.90	57.55	22.44	57.15	266.00	871.48	1204.78	7.61	I
9	510.10	5.55	2.24	51.03	4.43	1236.57	1199.41	7.68	II
10	386.50	61.28	25.28	57.30	334.88	827.02	1296.58	7.83	I
11	385.60	10.28	2.49	31.86	2.46	961.48	913.43	7.71	III
12	439.10	40.40	17.50	60.88	235.15	943.55	1264.81	7.79	I
13	447.10	42.32	12.84	60.88	193.66	952.65	1233.13	8.10	I
14	379.20	52.91	19.84	57.30	214.41	861.63	1154.48	7.94	I
15	385.20	67.82	18.70	50.98	263.54	931.43	1251.96	7.90	I
16	548.10	14.52	0.00	70.10	52.21	1364.10	1366.98	8.83	II
17	421.10	69.88	14.96	54.17	285.71	949.37	1323.46	8.33	I
18	423.80	59.98	21.95	60.69	225.53	927.80	1275.77	7.14	I
19	423.80	58.94	19.44	60.69	193.31	937.40	1237.14	7.21	I
20	407.56	21.28	33.02	56.60	269.60	784.43	1188.96	7.16	I
21	410.50	49.00	4.35	55.61	265.28	803.08	1194.48	7.80	I
22	390.38	23.10	25.01	59.50	263.42	982.86	1252.84	7.14	I
23	533.17	16.50	10.01	57.80	232.96	1016.21	1358.55	7.21	I
24	327.72	3.46	2.10	24.79	22.22	920.08	840.33	7.16	III
25	386.50	51.10	19.00	64.70	261.00	932.20	1279.00	7.70	I
26	419.35	51.10	19.10	59.50	229.00	1016.21	1286.16	7.68	I
27	537.63	15.20	9.84	59.30	231.00	940.00	1322.97	7.75	I
28	402.60	0.96	6.98	54.40	225.00	736.00	1057.94	7.80	III
29	407.02	19.40	7.06	0	251.00	859.20	1144.68	7.40	III
30	423.52	8.81	2.37	47.50	293.00	820.00	1185.20	8.12	III
31	453.40	40.61	11.08	58.32	278.72	974.18	1332.33	7.96	I
32	492.34	16.58	5.80	146.08	7.82	1064.02	1200.63	6.46	II
33	436.77	4.02	58.52	97.39	271.24	899.00	1117.44	6.65	I
34	187.56	12.87	10.21	9.92	54.54	555.19	552.70	6.87	III
35	587.60	6.09	3.08	64.29	17.03	1379.25	1385.57	8.31	II
36	398.30	33.91	31.79	162.47	325.11	580.49	1256.71	8.33	I
37	562.80	20.55	0.00	87.48	4.93	1429.28	1405.96	8.11	II
38	381.70	45.22	18.70	54.67	241.37	898.37	1190.85	7.83	I
39	346.10	55.49	2.26	54.67	246.30	879.39	1144.51	7.80	I
40	366.10	59.60	24.93	57.15	266.00	874.51	1211.04	7.72	I
41	337.00	62.94	14.78	58.94	209.26	835.07	1103.43	8.00	I
42	225.14	15.20	8.07	27.30	70.60	594.00	643.31	7.50	III

The primary water type in the uppermost Quaternary gravel layer pore aquifer is pore water, which is typically fresh. The main chemical constituents include bicarbonate (HCO₃⁻), calcium ions (Ca²⁺), and magnesium ions (Mg²⁺). The water is classified as hard or moderately hard, with a high degree of hardness. The thickness of the aquifer varies significantly, ranging from a few meters to tens of meters. The aquifer exhibits strong permeability and water storage capacity, with recharge primarily occurring through rainfall and river water. The water level is highly influenced by seasonal changes.

Next is the Permian clastic fissure water-bearing rock system, where fissure water is the predominant water type. The water quality is more complex, with chemical constituents such as sulfate (SO₄²⁻), calcium ions (Ca²⁺), and magnesium ions (Mg²⁺). The water is hard and mineralized, typically classified as hard or very hard. The thickness of the aquifer is substantial, generally ranging from tens to hundreds of meters, with a dominant composition of clastic rocks, such as sandstones and shales. The degree of fissure development significantly influences the permeability and water storage capacity of this aquifer.

Following this, the Upper Carboniferous Taiyuan Formation limestone karst fissure aquifer primarily contains karst fissure water, which typically has relatively clean water quality. The main chemical components are bicarbonate (HCO₃⁻), calcium ions (Ca²⁺), and magnesium ions (Mg²⁺). The water is hard, usually classified as hard or very hard. The thickness of the aquifer varies, generally between tens and hundreds of meters, with limestone being the predominant rock type. The well-developed karst system, along with the extent of fissure and cave development, significantly affects the permeability and water storage capacity. Recharge conditions are favorable, mainly through precipitation, surface water, and neighboring aquifers.

Finally, the Middle Ordovician karst fissure aquifer is characterized by karst fissure water, usually of relatively clean quality. The main chemical constituents are bicarbonate (HCO₃⁻), calcium ions (Ca²⁺), and magnesium ions (Mg²⁺). The water is typically hard or very hard, with thickness varying between tens and hundreds of meters. The aquifer exhibits well-developed karst, with permeability and water storage capacity influenced by the degree of fissure and cave development. Recharge is also facilitated through precipitation, surface water, and neighboring aquifers.

**Fig. 4:**
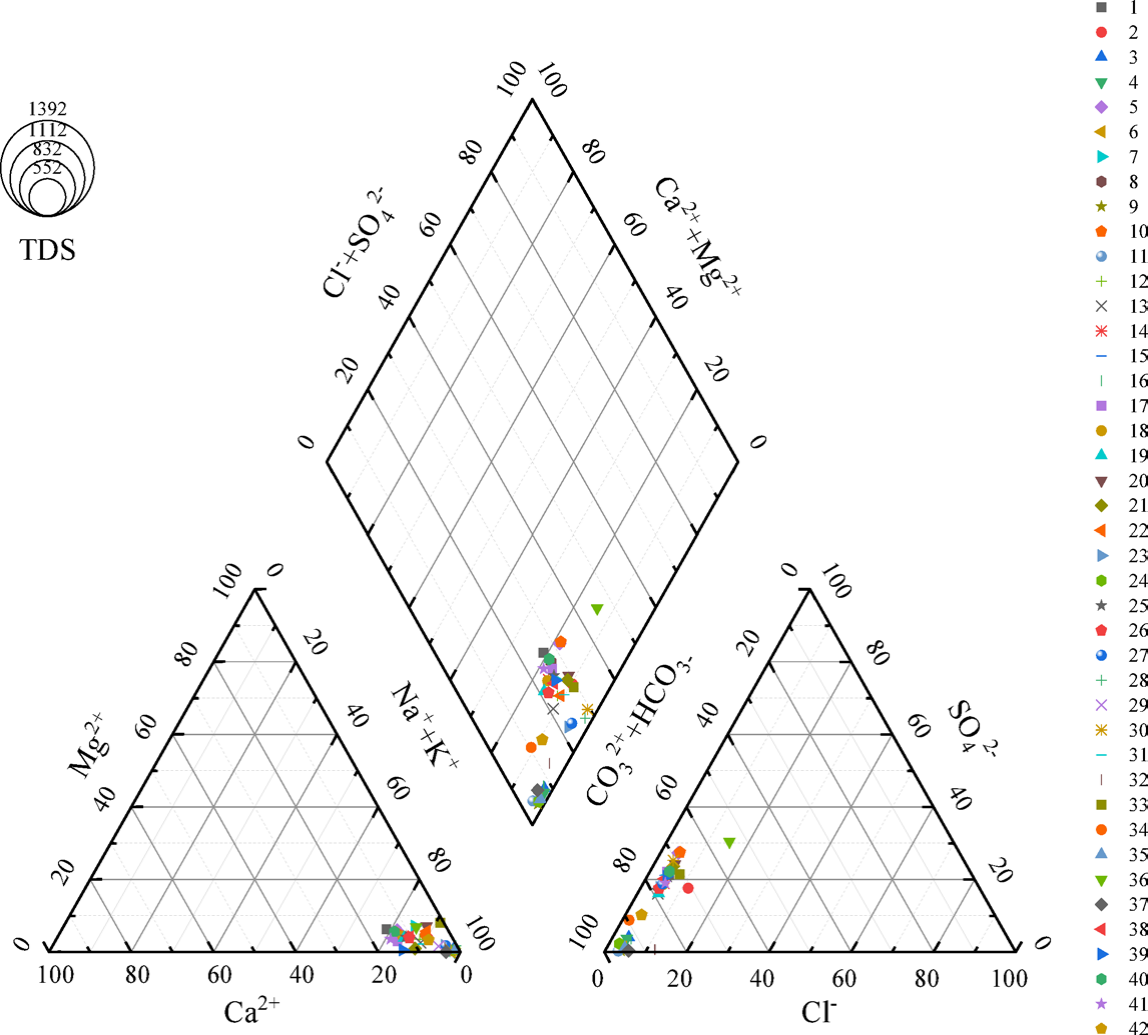
Piper’s trilinear diagram of water quality of water samples.

Based on the analysis of 42 groups of old void water samples from the Hanzui Coal Mine in Shanxi Province, the water samples are predominantly distributed across the Quaternary gravel layer pore-dividing aquifer, the Permian clastic rock fracture water-bearing rock series, and the Middle Ordovician karst fractured aquifer^43^. Building on this analysis, the fuzzy C-means (FCM) algorithm is applied to cluster the water source data. The optimal number of clusters is determined using the elbow method and the DB index. Subsequently, the cluster centers are established based on the weighted calculation of various chemical indicators, and the water source types are classified into three categories (see Table 1) A Piper trilinear plot (Fig. 4) was constructed using the 42 water sample data sets to provide a summary of the distribution of chemical features in the data. Subsequently, 29 of the 42 data sets were designated as the training set, while the remaining 13 sets were used as the prediction set. The datasets were preprocessed using chemical features, including K^+^+Na^+^,Ca^2+^,Mg^2+^,Cl^-^,SO_4_^2-^,and HCO_3_ as discriminators. The preprocessed data were then utilized for training the decision tree model.

## Establishment and validation of the FPS-DT model

### Fuzzy C-mean clustering algorithm

The chemical composition of water sources in the study area is highly complex, with limited differentiation between water types, necessitating a rigorous and systematic approach to data analysis. To ensure the reliability of subsequent modeling, raw data were first preprocessed by removing or imputing missing values using mean substitution. Outliers were identified and manually adjusted based on observed data patterns to prevent their undue influence on model performance. Subsequently, Z-score normalization was applied to standardize feature scales and eliminate dimensional disparities among variables.

**Fig. 5:**
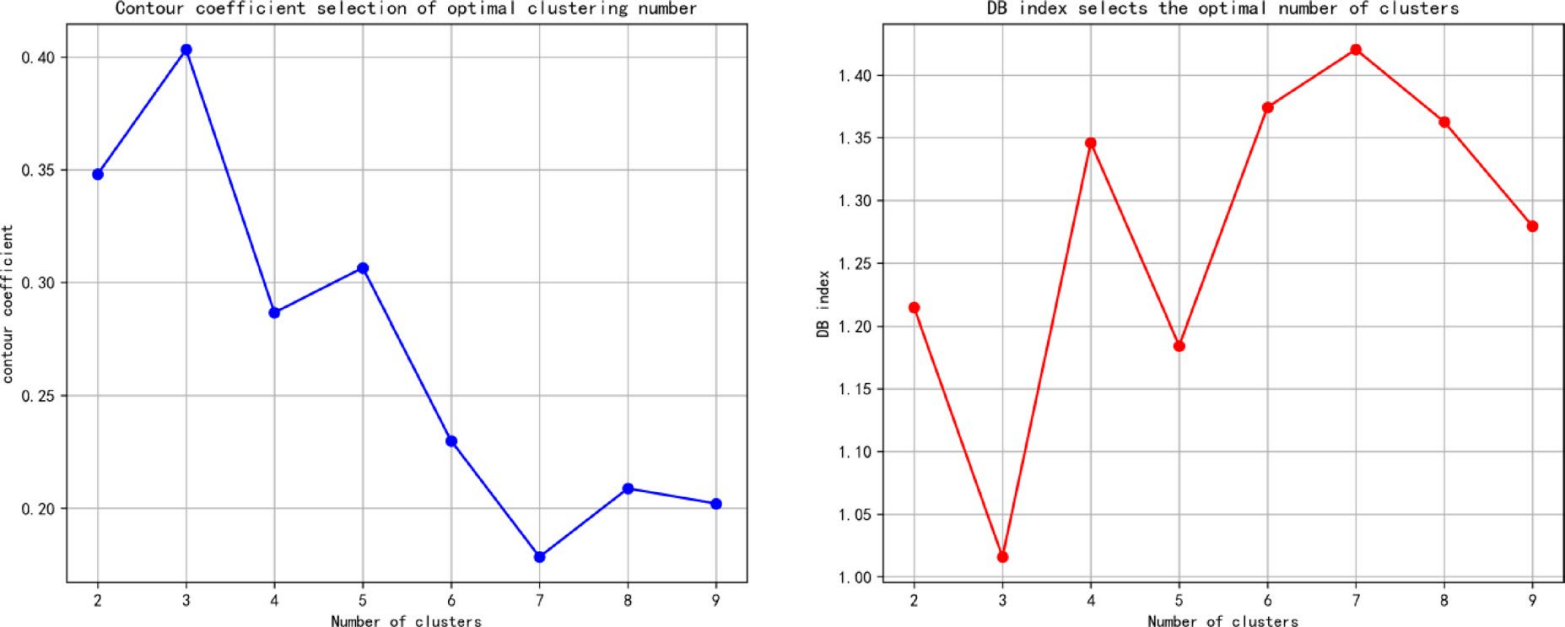
Optimal cluster number assessment diagram.

**Fig. 6:**
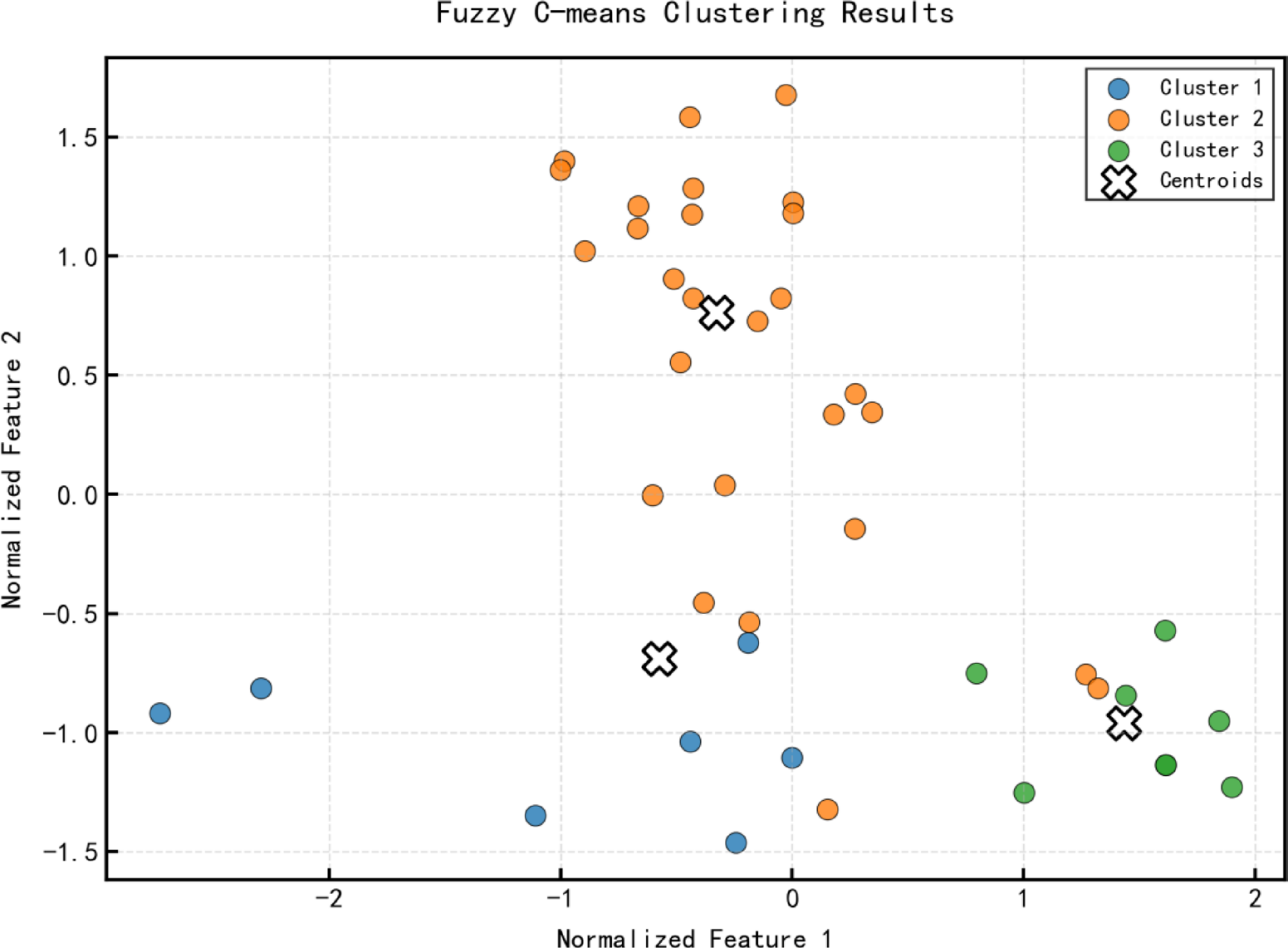
Clustering results.

Following data preprocessing, To determine the optimal number of clusters, the clustering performance is evaluated using both the Silhouette Coefficient and the Davies–Bouldin Index. The evaluation results suggest that three clusters yield the most appropriate partitioning for the given dataset. As illustrated in Fig. 5,corresponding to the highest silhouette score and the lowest Davies–Bouldin index. Based on these findings, the fuzzy C-means (FCM) clustering algorithm was employed to categorize the water source data, with cluster membership determined according to Eq. (1). The clustering results, presented in Fig. 6, show that the data were effectively partitioned into three distinct classes.

The resulting class labels derived from the FCM clustering were then used to construct the classification dataset for subsequent model development. This approach not only enhances the interpretability and granularity of hydrochemical data classification but also provides a robust foundation for the development of high-accuracy predictive models, thereby ensuring both scientific rigor and practical applicability.

### Principal component analysis

Based on the above clustering analysis results, the clustered data were used as the database for model construction in this study. The data analysis revealed that the major cations were K⁺, Na⁺, Ca²⁺, and Mg²⁺, while the major anions included Cl⁻, SO₄²⁻, and HCO3⁻. These chemical components are widely distributed across the study area and play a decisive role in determining the chemical type of groundwater sources.

To construct the classification model, 29 groups were randomly selected from 42 groups of field-collected data as the training set, with the remaining 13 groups serving as the prediction set. To enhance the efficiency of data analysis, principal component analysis (PCA) was applied for dimensionality reduction, thereby extracting key features from the chemical data. The model training was performed using the decision tree algorithm available in Python’s sklearn library. Before training, the sample set was balanced using the SMOTE oversampling technique to address the category imbalance problem, ensuring a more balanced representation during training.This approach not only captures the core information of the chemical features of water sources but also enhances the robustness and prediction accuracy of the model through effective training strategies.

The use of PCA helps in simplifying complex datasets by reducing the number of variables while retaining crucial information, and SMOTE ensures that the model is trained on a more balanced dataset. This method provides a scientific basis for classifying complex water sources based on their chemical characteristics.The data derived from the clustering results are subsequently subjected to Principal Component Analysis (PCA), which requires all variables to be on a consistent scale. Therefore, the data are standardized according to the method specified in Eq. (4). The standardized data are presented in Table 2.

**Table 2:** Normalised training data.

number	K^+^+Na^+^	Ca^2+^	Mg^2+^	Cl^−^	SO_4_^2−^	HCO3-+CO_3_^2−^	TDS	PH
1	-0.98	1.40	1.18	-0.01	0.54	-0.46	-0.19	0.42
2	0.27	-0.14	0.38	2.78	0.27	-0.35	0.57	0.76
3	1.61	-1.14	-1.00	-0.14	-1.24	1.85	0.98	0.44
4	1.61	-1.14	-1.23	-0.38	-1.31	1.55	0.68	0.50
5	-0.43	1.17	1.34	-0.25	1.40	-0.50	0.57	0.46
6	1.84	-0.95	-1.35	-0.16	-1.61	2.08	1.10	0.99
7	-0.60	0.00	1.61	-0.06	0.73	0.03	0.23	-1.26
8	-0.67	1.12	0.79	-0.14	0.70	-0.41	0.01	-0.20
9	1.00	-1.25	-1.13	-0.34	-1.75	1.39	-0.02	-0.05
10	-0.43	1.29	1.06	-0.13	1.35	-0.62	0.53	0.27
11	-0.44	-1.04	-1.11	-0.99	-1.76	0.04	-1.63	0.01
12	0.18	0.33	0.32	-0.01	0.42	-0.05	0.35	0.18
13	0.27	0.42	-0.13	-0.01	0.03	-0.01	0.18	0.84
14	-0.51	0.90	0.54	-0.13	0.22	-0.45	-0.27	0.50
15	-0.44	1.58	0.43	-0.35	0.68	-0.11	0.28	0.42
16	1.44	-0.84	-1.35	0.30	-1.30	2.01	0.93	2.39
17	-0.03	1.68	0.08	-0.24	0.89	-0.02	0.69	1.33
18	0.00	1.23	0.74	-0.02	0.33	-0.13	0.42	-1.20
19	0.00	1.18	0.50	-0.02	0.02	-0.08	0.20	-1.05
20	-0.18	-0.54	1.80	-0.16	0.74	-0.83	-0.07	-1.15
21	-0.15	0.73	-0.93	-0.19	0.70	-0.74	-0.04	0.21
22	-0.38	-0.45	1.03	-0.06	0.68	0.14	0.29	-1.20
23	1.27	-0.75	-0.39	-0.12	0.39	0.31	0.88	-1.05
24	-1.11	-1.35	-1.15	-1.23	-1.58	-0.17	-2.04	-1.15
25	-0.43	0.82	0.46	0.12	0.66	-0.11	0.43	-0.01
26	-0.05	0.82	0.47	-0.06	0.36	0.31	0.47	-0.05
27	1.32	-0.81	-0.41	-0.07	0.38	-0.07	0.68	0.10
28	-0.24	-1.46	-0.68	-0.23	0.32	-1.07	-0.81	0.21
29	-0.19	-0.62	-0.68	-2.06	0.56	-0.47	-0.32	-0.64
30	0.00	-1.10	-1.12	-0.46	0.96	-0.66	-0.10	0.88
31	0.35	0.34	-0.29	-0.10	0.82	0.10	0.74	0.54
32	0.80	-0.75	-0.80	2.85	-1.71	0.54	-0.01	-2.64
33	0.15	-1.32	2.32	1.22	0.75	-0.27	-0.48	-2.24
34	-2.73	-0.92	-0.38	-1.73	-1.28	-1.96	-3.67	-1.77
35	1.90	-1.23	-1.05	0.10	-1.63	2.09	1.04	1.29
36	-0.29	0.04	1.68	3.41	1.26	-1.83	0.31	1.33
37	1.61	-0.57	-1.35	0.88	-1.74	2.33	1.15	0.86
38	-0.48	0.55	0.43	-0.22	0.47	-0.27	-0.06	0.27
39	-0.90	1.02	-1.13	-0.22	0.52	-0.37	-0.33	0.21
40	-0.66	1.21	1.03	-0.14	0.70	-0.39	0.05	0.04
41	-1.00	1.36	0.06	-0.08	0.17	-0.58	-0.56	0.63
42	-2.30	-0.81	-0.58	-1.14	-1.13	-1.77	-3.16	-0.43

To analyze the standardized data, it is essential to calculate its covariance matrix, which provides a visual representation of the correlations between the variables. As shown in Table 3, the correlation coefficients between SO₄²⁻ and Mg²⁺, K⁺ + Na⁺ and TDS, Ca²⁺ and SO₄²⁻ are all greater than 0.6, indicating a relatively high reproducibility among the samples. Therefore, directly constructing the water identification model using the eight variables may significantly impact the accuracy of the model due to the strong correlations. Consequently, it is necessary to extract the principal components by evaluating their cumulative contribution in order to reduce the dimensionality of the data.

**Table 3:** Matrix of correlation coefficients for water chemical constituents.

correlation	K^+^+Na^+^	Ca^2+^	Mg^2+^	Cl_−_	SO_4_^2−^	HCO_3_-+CO_3_^2−^	TDS	PH
K^+^+Na^+^	1.00							
Ca^2+^	-0.35	1.00						
Mg^2+^	-0.37	0.48	1.00					
Cl_−_	0.31	0.03	0.28	1.00				
SO_4_^2−^	-0.32	0.61	0.70	0.10	1.00			
HCO_3_-+CO_3_^2−^	0.84	-0.30	-0.48	0.07	-0.57	1.00		
TDS	0.78	0.21	0.07	0.41	0.22	0.61	1.00	
PH	0.33	0.17	-0.29	0.07	0.02	0.30	0.42	1.00

The eigenvalues are derived from the computed covariance matrix Σ, which captures the inter-variable relationships in the standardized dataset. Subsequently, principal components are selected by identifying the eigenvectors corresponding to the largest eigenvalues. The contributions of each principal component and their associated eigenvalues are presented in Table 4.

A common approach is to use the cumulative variance contribution to determine how many principal components should be retained. According to Table 4, the first four principal components (Y₁ through Y₄) account for a cumulative variance of 91%, indicating that these components effectively capture the essential information from the original dataset without introducing significant error.

**Table 4:** Contribution of each principal component and eigenvalue.

Principal Component	Y1	Y2	Y3	Y4	Y5	Y6	Y7	T8
Variance Contribution Ratio	0.41	0.28	0.14	0.08	0.05	0.03	0.01	0
Cumulative Contribution Ratio	0.41	0.69	0.83	0.91	0.96	0.99	1	1
Eigenvalue	3.32	2.31	1.15	0.67	0.44	0.25	0.05	0.01

After the above analysis, four new principal components—PC1 through PC4—have been extracted, which effectively capture the key information from the previous eight indicators. The expressions for these four principal components are as follows:

11 $$\begin{gathered} \:{\text{PC}}1 = 0.497 \times \:\left( {{\text{Y}}1} \right) - 0.278 \times \:\left( {{\text{Y}}2} \right) - 0.374 \times \:\left( {{\text{Y}}3} \right) + 0.082 \times \:\left( {{\text{Y}}4} \right) \hfill \\ \: - 0.360 \times \:\left( {{\text{Y}}5} \right) + 0.511 \times \:\left( {{\text{Y}}6} \right) + 0.297 \times \:\left( {{\text{Y}}7} \right) + 0.224 \times \:\left( {{\text{Y}}8} \right) \hfill \\ \end{gathered}$$

12 $$\begin{gathered} PC2 = - 0.218 \times \:\left( {Y1} \right) - 0.394 \times \:\left( {Y2} \right) - 0.348 \times \:\left( {Y3} \right) - 0.370 \times \:\left( {Y4} \right) \hfill \\ \: - 0.425 \times \:\left( {Y5} \right) - 0.072 \times \:\left( {Y6} \right) - 0.538 \times \:\left( {Y7} \right) - 0.255 \times \:\left( {Y8} \right) \hfill \\ \end{gathered}$$

13 $$\begin{gathered} PC3 = 0.148 \times \left( {Y1} \right) - 0.389 \times \left( {{\text{Y}}2} \right) + 0.316 \times \left( {{\text{Y}}3} \right) + 0.573{\text{*}}\left( {{\text{Y}}4} \right) \hfill \\ - 0.096 \times \left( {{\text{Y}}5} \right) - 0.011 \times \left( {Y6} \right) + 0.0 \times \left( {{\text{Y}}7} \right) - 0.624 \times \left( {{\text{Y}}8} \right) \hfill \\ \end{gathered}$$

14 $$\begin{gathered} PC4 = - 0.178 \times \:\left( {Y1} \right) - 0.118 \times \:\left( {Y2} \right) - 0.191 \times \:\left( {Y3} \right) + 0.644 \times \:\left( {Y4} \right) \hfill \\ \: - 0.144 \times \:\left( {Y5} \right) - 0.323 \times \:\left( {Y6} \right) - 0.266 \times \:\left( {Y7} \right) + 0.553 \times \:\left( {Y8} \right) \hfill \\ \end{gathered}$$

After selecting the appropriate key variables through Principal Component Analysis (PCA), the next step is to construct a decision tree model for water source identification.

### The SMOTE algorithm

**Fig. 7:**
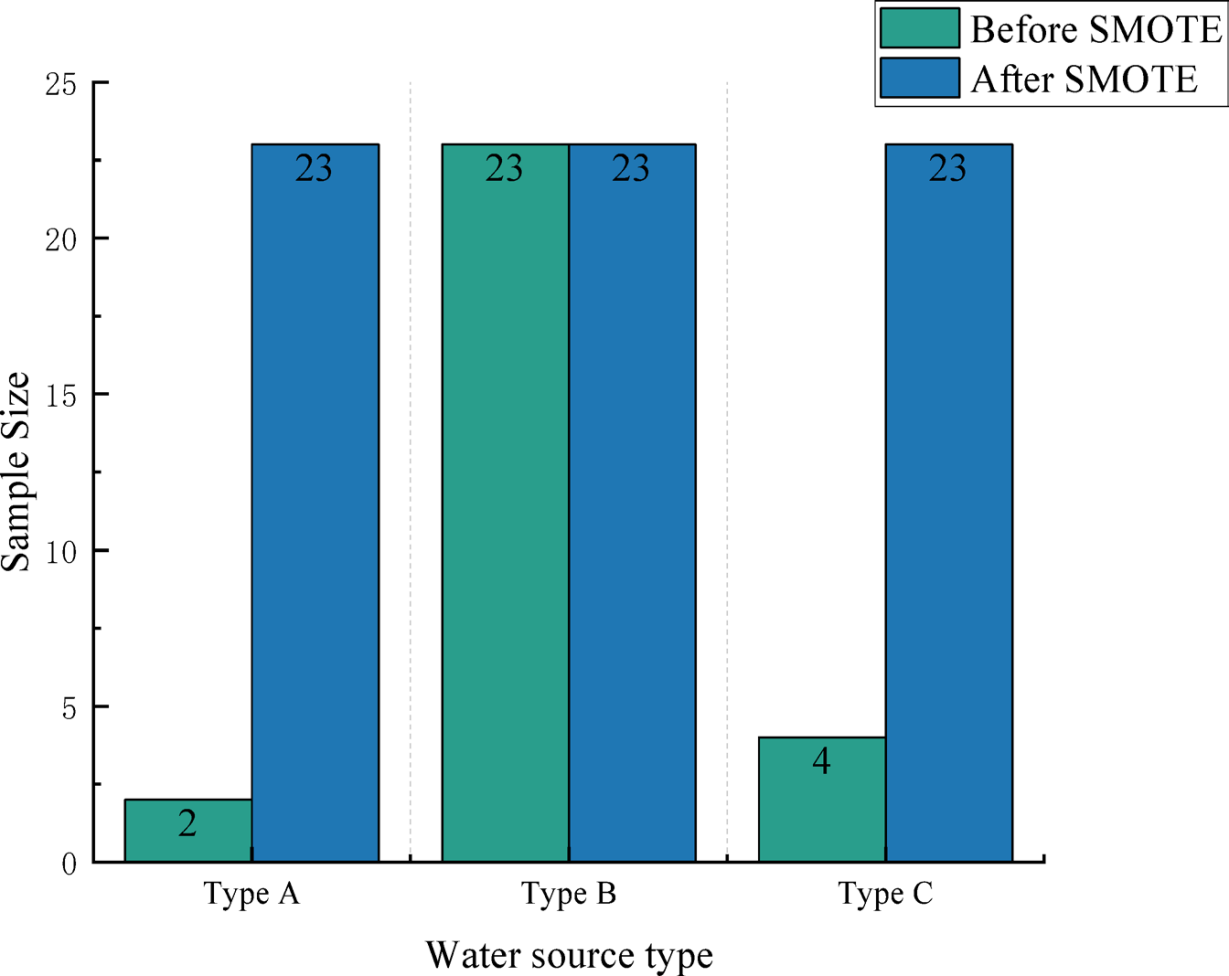
Comparison of data before and after SMOTE oversampling.

As shown in Table 1, the distribution of water samples in this study is highly uneven, with significant disparities in the number of samples across different water types. This imbalance adversely affects the accuracy of the trained decision tree model, leading to diminished classification performance. To address this issue, the Synthetic Minority Over-sampling Technique (SMOTE) is employed to augment the dataset post-Principal Component Analysis (PCA). By generating synthetic samples, SMOTE increases the representation of underrepresented water types, thereby mitigating class imbalance and enhancing the classification accuracy of the model. A sample is randomly selected from the minority class, and for each selected minority class sample, its k nearest neighbors within the same class are identified. In this study, the value of k is set to 3, meaning that the three nearest neighbor samples are used when generating synthetic samples. To ensure consistency across runs, the random seed is fixed at 6. Through the application of the SMOTE algorithm, the 29 samples in the training set are increased to 65. A comparison of the class distribution of sample data before and after applying the oversampling technique is presented in Fig. 7, illustrating the effectiveness of SMOTE in balancing the minority and majority classes. These 65 augmented samples are then utilized as the training set for constructing the decision tree model.

### FPS-DT modelling

**Fig. 8:**
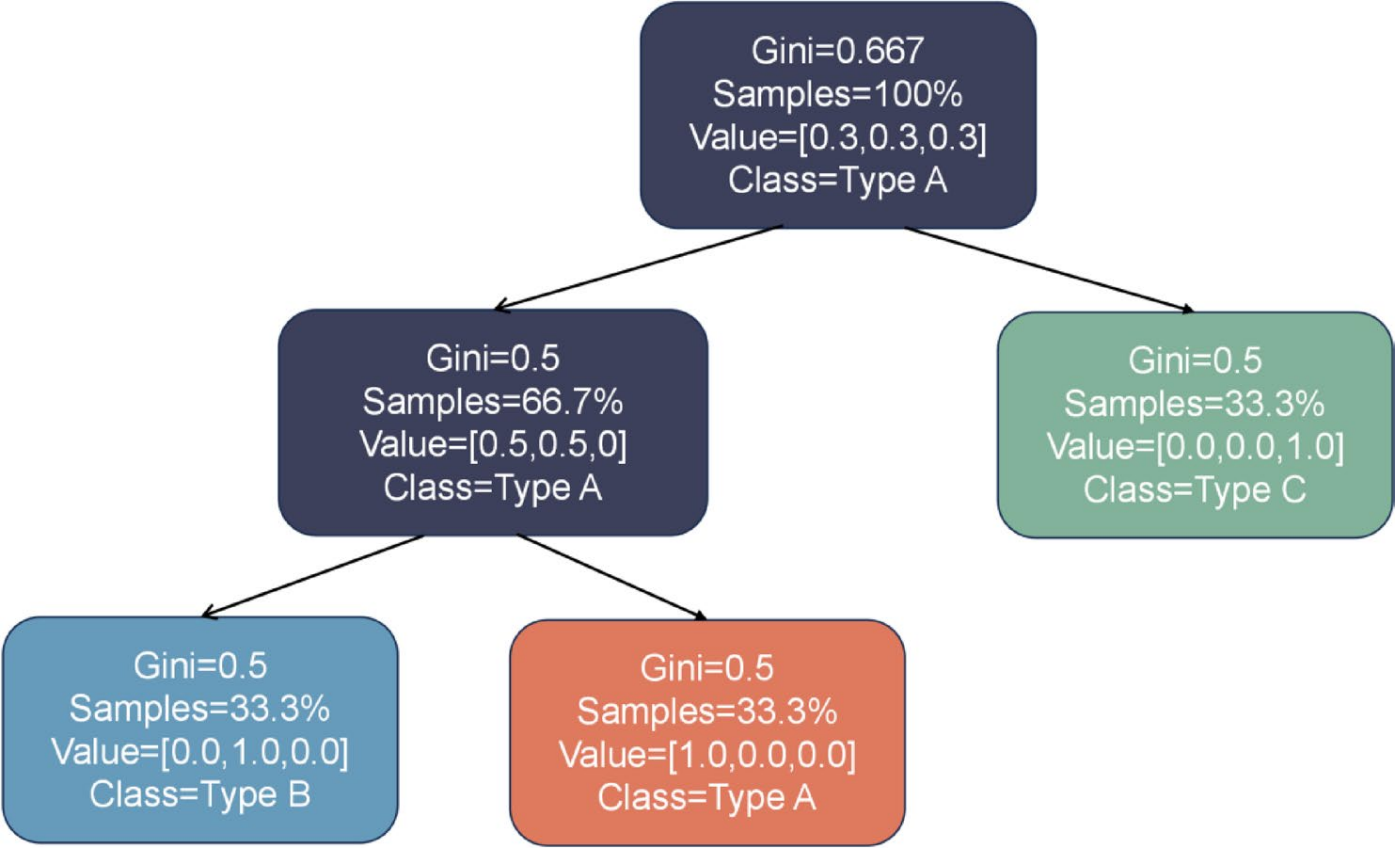
Decision Tree.

The dataset prepared above serves as the training sample for the model. A decision tree is constructed using the DecisionTreeClassifier from Python’s scikit-learn library, with PC1, PC2, PC3, and PC4 as input features. Prior to constructing the decision tree, the dataset is partitioned into training and validation sets. The decision tree undergoes pre-pruning, and its hyperparameters are optimized using grid search with 5-fold cross-validation. This approach ensures that the model’s performance is evaluated on different training and validation datasets, thereby reducing the risk of overfitting. The recognition accuracy is employed as the evaluation metric for the model. Finally, the decision tree model is built based on the optimal parameters derived from the grid search. The decision tree model constructed in this study employs the Classification and Regression Trees (CART) algorithm, utilizing Gini impurity as the criterion for feature selection. A lower Gini impurity indicates a more distinct classification of the samples. The training set comprises 65 samples, augmented through the Synthetic Minority Oversampling Technique (SMOTE) to address class imbalance. Modelling was carried out using the procedure described above:

1. Construct a root node containing all training datasets and configure its parameters: Maximum depth of 4,Minimum split node size of 2,Minimum leaf node size of 1,Complexity pruning set to 0.03.After completing the pre-pruning, select an optimal feature derived from Principal Component Analysis (PCA) and calculate its Gini coefficient using Eq. (4). Using this optimal feature as a basis, partition the training dataset into multiple subsets. Each subset will achieve the best possible classification results given the current conditions.
2. If all subsets achieve basic correct classification, the process of constructing leaf nodes is complete. However, if there are still subsets that cannot be correctly classified, new optimal features need to be selected for these subsets. The division process continues, and corresponding nodes (branches) are constructed recursively. This process repeats itself until all subsets of the training data can be classified essentially correctly or until no suitable features remain for further selection.
3. When all subsets have been assigned to leaf nodes and the Gini coefficient reaches zero (i.e., each subset contains clear classification labels), the decision tree model is considered fully trained (Fig. 8). Once the training of the decision tree model is completed, data from the prediction set is input into the model for recognition testing. The results obtained from the decision tree are then output using Python’s visualization module.

### Analysis of discriminatory results

To evaluate the performance of the constructed decision tree model, classification accuracy was assessed using the ‘metrics’ module from the scikit-learn library. A classification report was generated to summarize key evaluation metrics, including precision, recall, and F1-score, as presented in Table 5. In addition, five-fold cross-validation was employed to estimate the model’s generalization ability. This method partitions the dataset into five subsets and iteratively uses four for training and one for validation, thereby providing a robust and unbiased estimate of predictive performance.

**Table 5:** Classification report.

	Precision/%	Recall/%	f1-score/%	Support/%	Accuracy/%
I	100	100	100	2	92
II	89	100	94	8	
III	100	67	80	3	

**Fig. 9:**
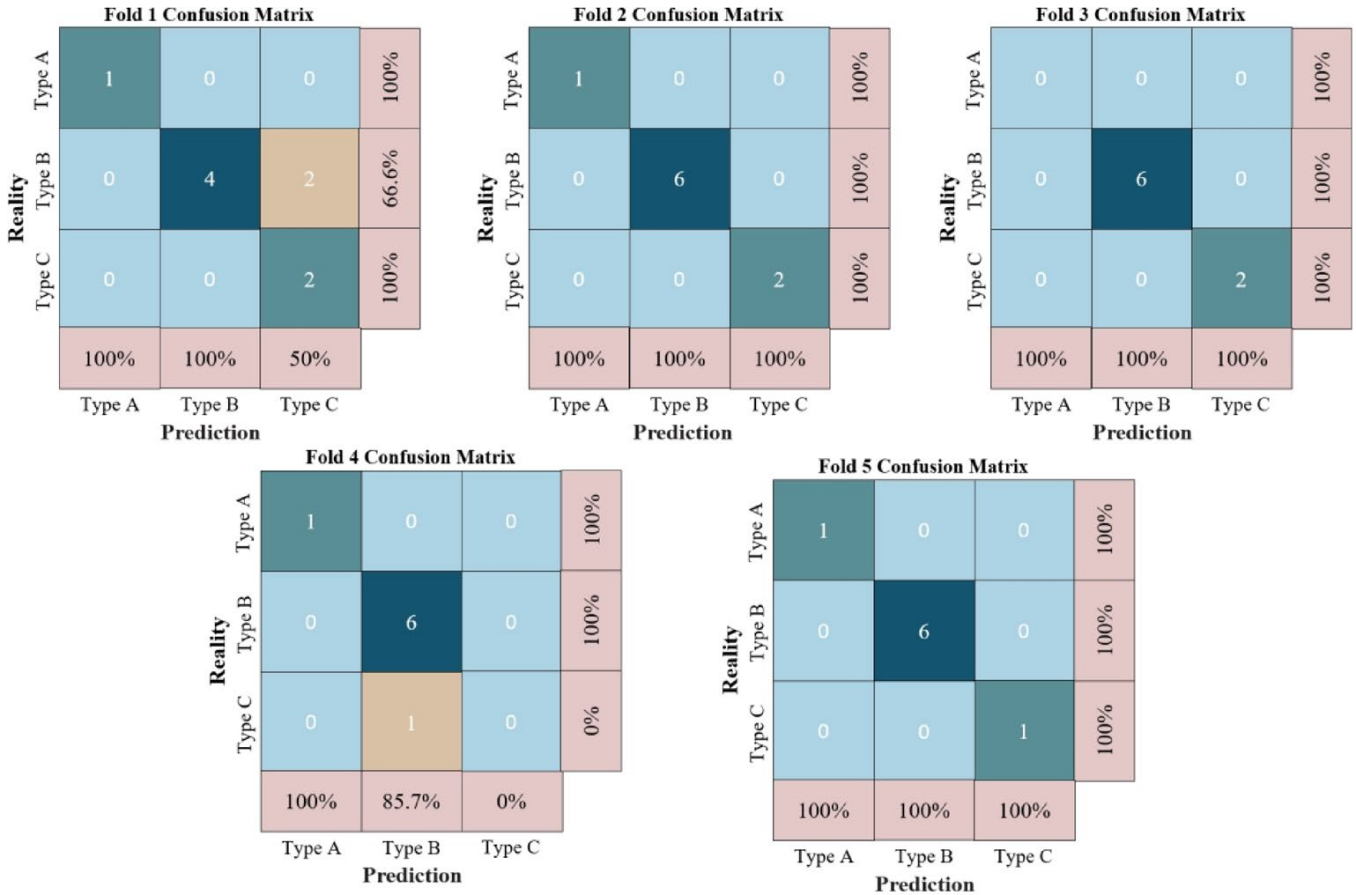
confusion matrix.

In order to further explain the behaviour of the model, the confusion matrix for each validation during the five-fold cross-validation was output (Fig. 9). In this matrix, the horizontal axis represents the predicted labels and the vertical axis represents the true labels. Diagonal entries correspond to correctly classified instances, while off-diagonal entries indicate misclassification. This visualisation helps to intuitively understand the model’s ability to classify in different water types and reveals potential weaknesses in specific category distinctions.

The classification report indicates that the model achieves a discrimination accuracy of 89% for Type II, with both a recall and F1 score of 100%, demonstrating a well-balanced performance between precision and recall. Additionally, the model exhibits exceptional performance for Type I, with perfect accuracy, recall, and F1 scores of 100%, highlighting its high precision in discriminating Type I. The F1 score, which integrates both precision and recall, is particularly useful for evaluating the model’s performance in cases of class imbalance. The formula is as follows:

15 $$F1=2 \times \frac{{{\text{precision}} \times {\text{recall}}}}{{{\text{precision}}+{\text{recall}}}}$$

Where precision is the accuracy of the current category and recall is the recall of the current category.

The model achieves an identification accuracy of 100% for Type III; however, the recall for Type III is relatively low. This discrepancy is likely due to the uneven distribution of samples, with a particularly low number of Type III samples in the test set. The insufficient sample size for Type III likely hinders the model’s ability to effectively identify this type. In conclusion, based on the old goaf water quality data from Hanzui Mine, the overall recognition accuracy of the model across all sample types reached 92%.

**Fig. 10:**
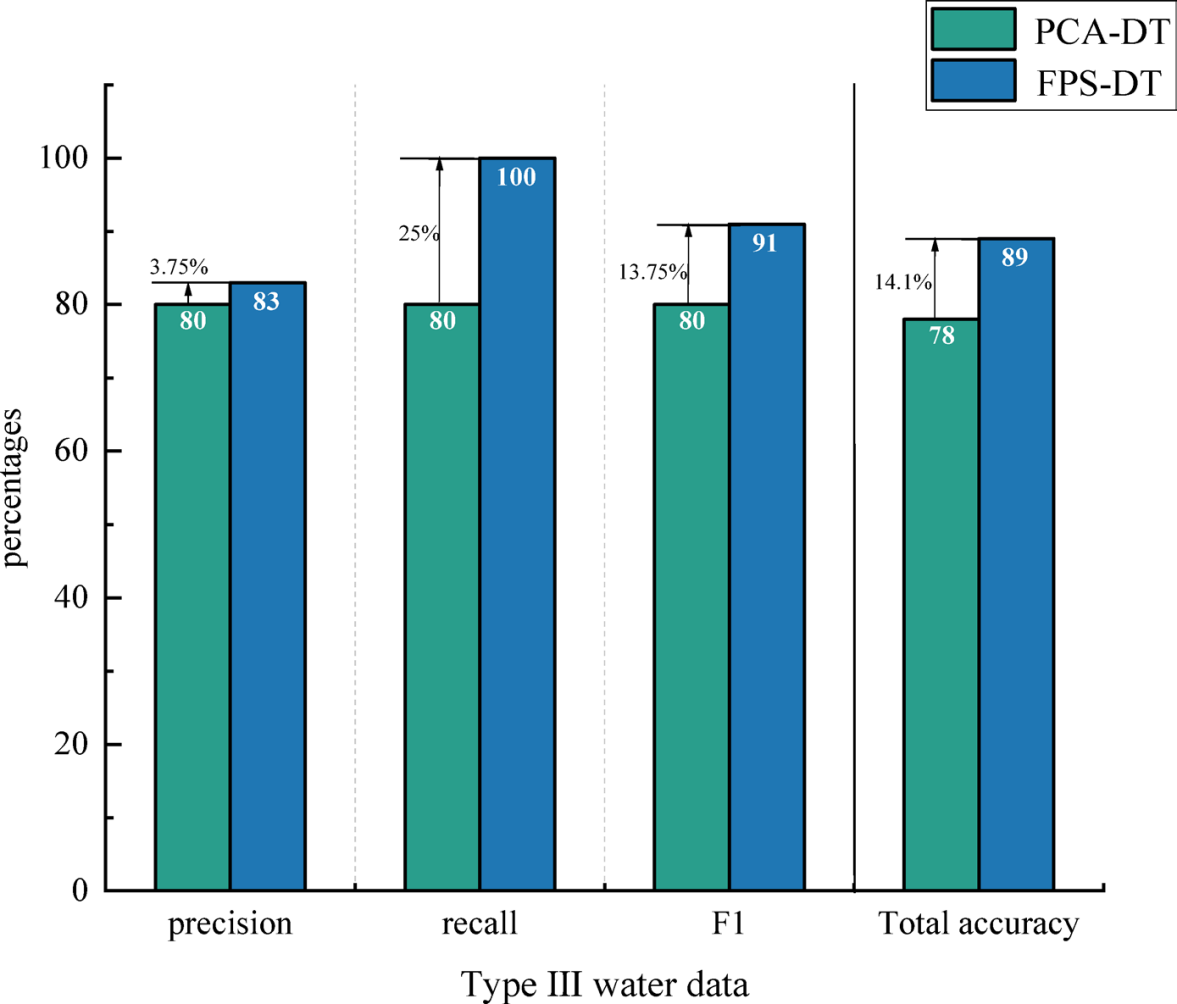
Comparison of FPS-DT and PCA-DT results graphs.

To further validate the accuracy and reliability of the FPS-DT model, identical data samples and software platforms were used in the experiments presented in this study. The parameters of the decision tree model were maintained in alignment with those used in the FPS-DT model. Following Principal Component Analysis (PCA) preprocessing, the data were not subjected to the SMOTE oversampling technique and were directly modeled and predicted using the decision tree model. Classification reports for both PCA and the decision tree were generated. Finally, a comparative analysis of the accuracy, recall, and F1 scores of the two models was conducted for the Type III classification task to evaluate their respective classification performance(Fig. 10).

In this study, the PCA combined with decision tree model (PCA-DT) and the FPS-DT model are compared and analyzed under the same dataset and parameter settings. The results indicate that the accuracy of the standard PCA-DT model is only 78%, while the FPS-DT model demonstrates significant improvements in accuracy, recall, and F1 score after incorporating the SMOTE oversampling technique. These findings suggest that the decision tree model, when combined with PCA for feature extraction and SMOTE for data augmentation, offers a more reliable method for identifying groundwater sources, with enhanced accuracy and applicability, thereby effectively addressing the needs of groundwater source identification.

## Conclusions

In this study, forty-two sets water samples were randomly selected and plotted on Piper trilinear diagrams to examine the hydrochemical characteristics of the water sample data, with the Hanzui Old Air Water quality serving as the study’s background. The chemical indicators, including Na^+^ + K^+^, Ca^2+^, Mg^2+^, Cl^−^, SO_4_^2−^, HCO_2_−, and CO_2_^2-^, constitute the predominant factors in these water sources, thereby defining the chemical composition of the water. These elements were utilized as key conditions for identifying sudden water sources. This study employs the Fuzzy C-Means (FCM) algorithm to perform clustering analysis of these elements, using the results as criteria for identifying potential water inrush sources. Additionally, Principal Component Analysis (PCA) is applied to process the data, with four principal components selected based on the cumulative variance contribution. This approach effectively reduces redundancy among the variables, enhancing the model’s efficiency. To address the issue of class imbalance in model training, the Synthetic Minority Over-sampling Technique (SMOTE) is applied to augment the minority class samples. A groundwater source identification model is then constructed using a decision tree based on the Classification and Regression Tree (CART) algorithm. The Gini index is utilized as the feature splitting criterion, and the feature with the smallest Gini-weighted value is selected as the root node of the tree. This process is iteratively repeated until a complete decision tree is formed. The Gini coefficient was used as the criterion for feature splitting, where the feature with the smallest Gini index value was selected as the root node of the decision tree. This process continued recursively until the decision tree was fully constructed. The experimental results demonstrate that the proposed method exhibits high recognition accuracy and strong potential for practical application. Furthermore, the model structure is simple and intuitive, making it easy to understand and interpret, with high readability. This method not only enhances safety in future mining operations by reducing the risk of sudden water-related accidents but also offers a novel and effective approach for groundwater source identification. Through precise data analysis and feature identification, this method holds significant promise for broader applications in hydrogeological research and related field.o understand and interpret, with high readability. This method not only enhances safety in future mining operations by reducing the risk of sudden water-related accidents but also offers a novel and effective approach for groundwater source identification. Through precise data analysis and feature identification, this method holds significant promise for broader applications in hydrogeological research and related field.

## Supplementary Information

Below is the link to the electronic supplementary material.

Supplementary Material 1

Supplementary Material 2

## Data Availability

All data generated or analyzed during this study are included in the published paper. The detailed data could be supplied on demand after corresponding author.
