# Supplementary figures and images for: Identification of water sources of mine water bursts based on the FPS-DT model
Source: Sci Rep. 2025 Jul 27;15:27327. doi: 10.1038/s41598-025-13301-y (PMC12301436; doi:10.1038/s41598-025-13301-y)

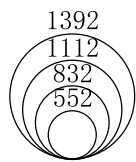

TDS

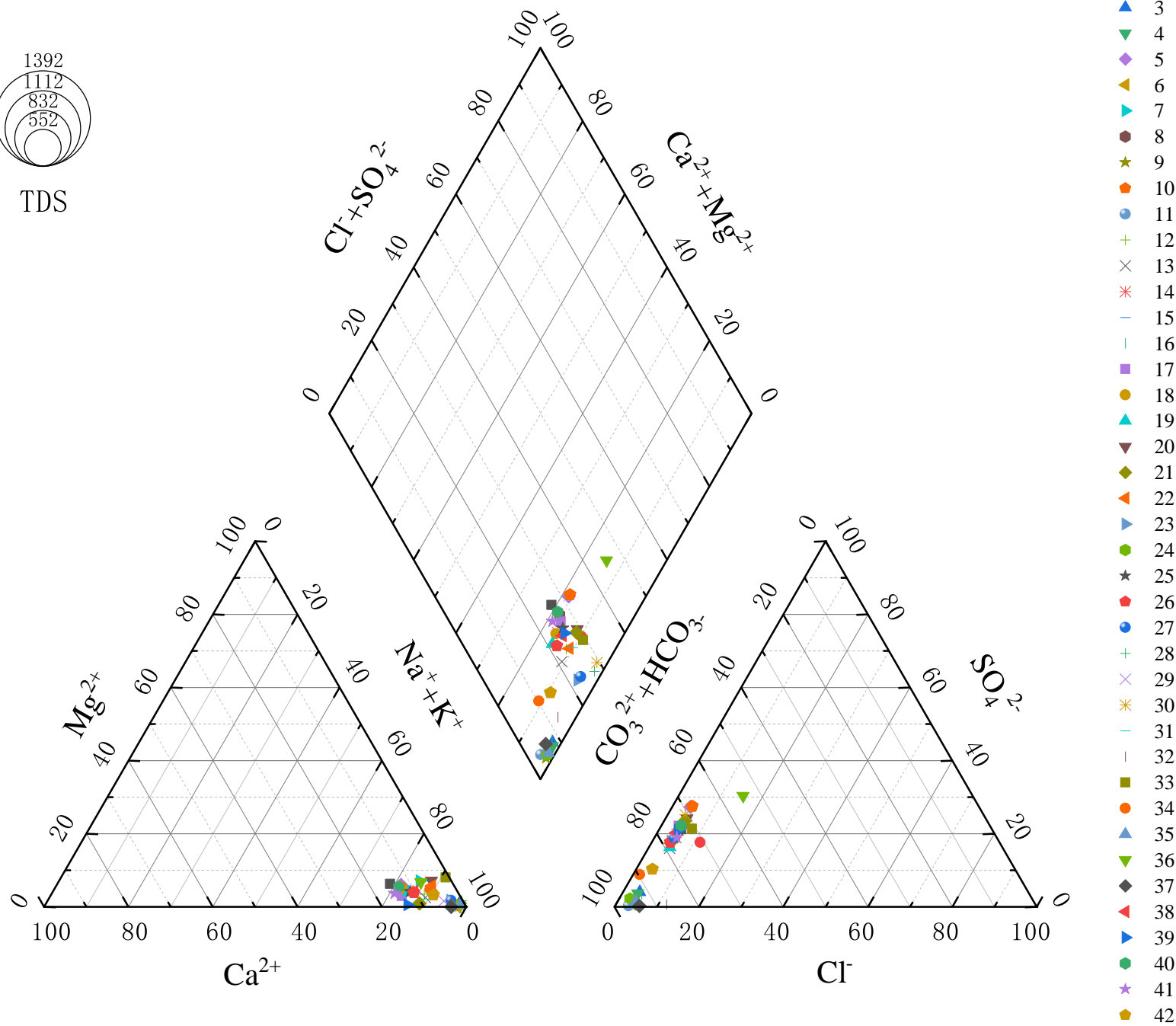

Supplement: Supplementary file 1 — Supplementary Material 1 [file 41598_2025_13301_MOESM1_ESM.pdf]
